# Supplementary material for: Phylogenetic Analysis of Pyruvate-Ferredoxin Oxidoreductase, a Redox Enzyme Involved in the Pharmacological Activation of Nitro-Based Prodrugs in Bacteria and Protozoa
Source: Biology (Basel). 2024 Mar 9;13(3):178. doi: 10.3390/biology13030178 (PMC10968658; doi:10.3390/biology13030178)
Supplement: Supplementary file 1 [file biology-13-00178-s001.zip › biology-2765984-supplementary.pdf]

Table S1

Gene and accession numbers of the redox enzymes and control proteins from the UniProt™ Protein Database

**Parasite**	**Enzyme**	**Gene number**	**Accession number**	**Redox drug**
G. duodenalis	PFOR	Q24982	AAA74894.1	Metronidazole
E. histolytica	PFOR	N9TJX2	ENY63830.1	Metronidazole
C. parvum	PFOR	A1DRF1	ABK91849.1	Nitazoxanide

Parasite	Control protein1	Gene/Accession number	Control protein 2	Gene/Accession number	Control protein 3	Gene/Accession number
G. duodenalis	GAPDH	**P53429**<br>AAB18421.1	TUB	A8BPC0<br>EDO78046.1	**RPB1**	**Q8MUU2**<br>AAM77743.1
E. histolytica	GAPDH	C4LVR9<br>BAN37492.1	TUB	P31017<br>AAA57315.1	**RPB1**	Q6IUR3<br>AAT40981.1
C. parvum	GAPDH	Q7YYQ9<br>BAJ77164.1	TUB	Q9UAC3<br>AAD20239.1	**RPB1**	**A0A7G2HJ78**<br>**CAD98371.1**

Table S2

List of protozoa showing how their lifecycle supports the horizontal acquisition of PFOR from bacteria based on the proximity hypothesis.

Protist	Life cycle	PFOR
**Blastocystis**	**Gastro-intestinal tract, facultative, strict anaerobe, direct vertebrate parasites**	+
Phytophthora	Free living fungal-like protists, plant parasites	
Pythium	Free living fungal-like protists, predominantly plant parasites	
Achlya	Free living fungal-like protists, plant parasites	
Thraustotheca	Free living fungal-like protists, plant parasites	
Theileria	Erythrocytes, insect-borne cattle parasite	
Babesia	Erythrocytes, insect-borne vertebrate parasites	
Plasmodium	Erythrocytes, insect-borne vertebrate parasites	
**Eimeria**	**Gastro-intestinal tract, direct vertebrate parasites**	?
**Cryptosporidium**	**Gastro-intestinal tract, direct vertebrate parasites**	+
Toxoplasma	Diverse cells, obligate intracellular vertebrate parasites	
Tetrahymena	Free living	
Paramecium	Free living	
Plasmodiophora	Free living	
Reticulomyxa	Free living	
Trypanosoma	Blood stream, insect-borne vertebrate parasites	
Leishmania	Macrophages, dendritic cells, insect-borne vertebrate parasites	
Naegleria	free-living amoeboflagellate, aerobic and anaerobic	
**Entamoeba**	**Gastrointestinal tract, anaerobe, direct vertebrate parasites**	+
Dictyostelium	Free living	
**Giardia**	**Gastrointestinal tract, anaerobe, direct vertebrate parasites**	+
**Tritrichomonas**	**Genito-urinary tract, anaerobe, direct vertebrate parasites**	+
**Trichomonas**	**Genito-urinary tract, anaerobe, direct vertebrate parasites**	+

Straminopiles | Alveolates | Rhizaria

Excavates | Amoebozoa | Metamonads
